# Central adrenal insufficiency: who, when, and how? From the evidence to the controversies – an exploratory review

**DOI:** 10.20945/2359-3997000000493

**Published:** 2022-06-27

**Authors:** Mariana Rechia Bitencourt, Rafael Loch Batista, Isabela Biscotto, Luciani R. Carvalho

**Affiliations:** 1 Universidade São Paulo Faculdade de Medicina Hospital das Clínicas São Paulo SP Brazil Unidade de Endocrinologia do Desenvolvimento, Disciplina de Endocrinologia, Departamento de Clínica Médica, Hospital das Clínicas, Faculdade de Medicina, Universidade São Paulo, São Paulo, SP, Brasil; 2 Instituto do Câncer do Estado de São Paulo São Paulo SP Brazil Instituto do Câncer do Estado de São Paulo, São Paulo, SP, Brasil; 3 Saúde de Juiz de Fora Faculdade de Ciências Médicas Juiz de Fora MG Brazil Faculdade de Ciências Médicas e da Saúde de Juiz de Fora (Suprema), Juiz de Fora, MG, Brasil

**Keywords:** Central adrenal insufficiency, secondary adrenal insufficiency, ACTH deficiency, cortisol replacement, glucocorticoid replacement

## Abstract

Central adrenal insufficiency (CAI) is a life-threatening disorder. This occurs when ACTH production is insufficient, leading to low cortisol levels. Since corticosteroids are crucial to many metabolic responses under organic stress and inflammatory conditions, CAI recognition and prompt treatment are vital. However, the diagnosis of CAI is challenging. This is not only because its clinical presentation is usually oligosymptomatic, but also because the CAI laboratory investigation presents many pitfalls. Thus, the clarification of when to use each test could be helpful in many contexts. The CAI challenge is also involved in treatment: Several formulations of synthetic steroids exist, followed by the lack of a biomarker for glucocorticoid replacement. This review aims to access all available literature to synthesize important topics about who should investigate CAI, when it should be suspected, and how CAI must be treated.

## INTRODUCTION

Central adrenal insufficiency (CAI) occurs when corticotropin-releasing hormone (CRH) or adrenocorticotropic hormone (ACTH) signaling cannot guarantee the proper quantity of adrenal androgens and glucocorticoids ( 1 ). Mineralocorticoids produced by the adrenal gland are regulated mainly by the renin-aldosterone system; therefore, their secretion is preserved in CAI.

The hypothalamic-pituitary-adrenal (HPA) axis controls cortisol production in response to light, stress, and many other inputs, including communication with the autonomic nervous system ( 2 , 3 ). This robust orchestration is responsible for the circadian rhythm ( 2 ). Glucocorticoids regulate the fundamental processes in the human body. The HPA axis is the main connector between the immune and endocrine systems ( 4 , 5 ). They link the endocrine, cardiovascular, and immune systems to ensure the correct response to inflammatory and immunological events ( 3 , 6 , 7 ). Therefore, a lack or excess of glucocorticoids impacts these physiological processes in many ways.

Evaluation of functional CAI is extremely important in various clinical and intensive care contexts, as an inadequate response of the axis interferes with the recovery from many diseases ( 3 ). CAI is a challenging condition because of its oligosymptomatic presentation or complicated laboratory confirmation ( 8 ). Unlike Addison’s disease, where ACTH excess can lead to skin hyperpigmentation, and mineralocorticoid deficiency, which causes postural hypotension, volume depletion, and hyperkalemia, in CAI, the absence of clinical signs may occur. These features are dependent on severity, time of onset, associated pituitary deficiencies, and clinical context. In newborns, ACTH deficiency may present as hypoglycemia, jaundice, seizures, and failure to thrive ( 9 ). In adults, most associated symptoms are nonspecific, ranging from anorexia, weakness, myalgia, and adynamia to nausea, vomiting, hypoglycemia, and hypotension during an adrenal crisis ( 10 , 11 ). The abnormal laboratory findings are highlighted in Table 1 . It is noteworthy to mention that CRH and ACTH absences could generate symptoms besides cortisol deficiency. These peptides have behavioral activities in anxiety, mood, locomotion, reward, and feeding ( 5 , 12 ) and increase sympathetic activation ( 1 ).

**Table 1:** Laboratory findings of hypocortisolism

Hyponatremia ( due to vasopressin increase)
Hypoglycemia
Normocytic anemia
Lymphocytosis
Eosinophilia
Hyperbilirubinemia (children)

Another relevant factor is the peripheral activity of corticosteroids in different tissues and their genomic and non-genomic effects. Some polymorphisms of glucocorticoid receptors have been described to be related to glucocorticoid syndromes of resistance or hypersensitivity ( 13 , 14 ). While genomic actions tend to be chronic and mediated by glucocorticoid receptors and their transcriptional processes, non-genomic processes involve signalization in the cell membrane with immediate reactions ( 15 ). Recently, research has expanded the knowledge about these non-genomic pathways, even though more studies could further elaborate knowledge on this topic.

## WHO?

### Incidence and etiology

CAI is classified as secondary or tertiary. The secondary causes are pituitary conditions, whereas the tertiary causes are hypothalamic disorders as well as CRH dysfunction. The annual incidence of CAI is 14-28 per 100,000 individuals ( 8 , 16 , 17 ). HPA inhibition by corticosteroid use is the most common cause of CAI ( 1 , 17 ). Corticosteroids are used worldwide for several medical conditions, but rational use is mandatory since corticosteroid misuse may have clinical consequences. However, the daily use of 20 mg or more of hydrocortisone or its equivalents (HCeq) for longer than 3 weeks might be related to ACTH suppression. This suppression by exogenous drugs is not exclusive to corticosteroid use; the abuse of opiates can also suppress ACTH once corticotroph inhibition is noted in 10%-20% of individuals using daily morphine-equivalent doses of 100 mg or more ( 18 , 19 ). In addition to exogenous corticosteroids, diverse conditions may also result in CAI ( Table 2 ). Traumatic brain injury (TBI) is related to hypopituitarism in the frequency range of 16% to 69% ( 20 ), which could be dependent on patient selection, evaluation during different times along the disease course, TBI severity, diagnostic criteria, dynamic stimulation testing methods, and diagnostic cut-off values ( 20 , 21 ). A meta-analysis including 66 studies (besides high heterogeneity index = I² >75%) showed that TBI was related to hypopituitarism (any pituitary axis) in a large range spanning 5% to 90%, but the prevalence of CAI due to TBI ranges from 7% to 13% ( 22 ). CAI is a common side effect of radiotherapy for intracranial tumors ( 23 ). Immunotherapy has been related to primary and central adrenal insufficiency due to adrenalitis and hypophysitis, respectively ( 24 ). Hypophysitis is more frequently associated with anti-CTLA4 drugs (1.5 % to 17%) than with anti-PD1 (0.5%-1.5%) or anti-PDL1(<0.1-0.2%)( 25 ). Primary adrenal insufficiency, although rare, was related to both drug classes, ranging from 0.8% to 1.6%of the frequency ( 25 ). However, since immunotherapy has emerged as a first-line therapy for several medical conditions, even this low frequency must not be ignored. A meta-analysis of 38 studies reported that patients who received anti-PD1 drugs had 0.29 less risk of developing any grade of hypophysitis than those who received ipilimumab(anti-CTLA4 drug), whereas the patients who received these two medications in combination had a 2.2 odds ratio to develop hypophysitis ( 26 ).

**Table 2:** Etiologies of central adrenal insufficiency

Iatrogenic			Procedure	
	Medication			Hypothalamic and pituitary surgery
		Glucocorticoids		Radiosurgery and radiotherapy
		Opiates		
		Megestrol acetate		
		Medroxyprogesterone		
		Immune checkpoint inhibitors		
Hypophysitis			Secondary hypophysitis	
	Infiltrative disease			Sarcoid granulomatosis with polyangiitis
		Hemochromatosis		Langerhans cell histiocytosis
		Amyloidosis		Infection
		PIT1-antibody		
	Primary hypophysitis			
		Lymphocytic		
		Granulomatous		
		Xanthomatous		
Vascular				
	Sheehan’s syndrome			
	Aneurysmal subarachnoid hemorrhage			
	Pituitary apoplexy			
	Arteritis			
Tumors				
	Pituitary abscess		Ependymoma	
	Pituitary adenoma		Glioma	
	Empty sella		Pinealoma	
	Parasellar tumors or cysts		Craniopharyngioma	
	Rathke’s cyst		Hypothalamic hematoma	
	Dermoid cyst		Gangliocytoma	
	Meningioma		Metastasis	
	Germinoma		Hematological malignancies (leukemias, lymphomas)	
	Chordoma			
Traumatic				
	Head injury			
	Perinatal trauma			
Genetic				
	Combined pituitary hormone deficiencies		Associated syndromes	
		*POUF1*		Septooptic dysplasia ( *HESX1* )
		*PROP1*		GH, TSH, ACTH deficiencies and cerebellar abnormalities ( *LHX4* )
	Isolated ACTH deficiency			Hypopituitarism and mental retardation ( *SOX3* )
		*TBX19 (TPIT)*		Holoprosencephaly and multiple midline defects ( *GLI2* )
		*POMC*		Anophthalmia, hypopituitarism, esophageal atresia ( *SOX2* )
		*PC1(PCSK1) – POMC* processing defect		CHARGE syndrome
				Prader-Willi syndrome
Idiopathic				

Overall, up to one-third of individuals with pituitary disease develop subsequent CAIs ( 27 , 28 ). Even high, the prevalence can be underestimated. A Brazilian retrospective study including 99 patients with hypopituitarism (53% of tumoral etiology) evidenced 82% of CAI prevalence ( 29 ). However, congenital causes of CAI are rare. Genetic causes of CAI include pathogenic mutations in genes encoding transcription factors related to pituitary development, such as GLI2, OTX2, HESX1, LHX3, LHX4, and PROP1, whose clinical presentations are variant degrees of hypopituitarism ( 1 ). Mutations in the TBX19 gene (previously described as TPIT) result in isolated ACTH deficiency, which is rare but presents with a recessive inheritance pattern ( 9 , 30 ).

## WHEN?

### Diagnostic approach

#### Basal cortisol measurement

The basal cortisol level cannot predict the response to ACTH ( 31 ). Thus, the use of basal cortisol to diagnose CAI requires care. In addition, there are many pitfalls in laboratory measurements of morning serum cortisol levels. Nonetheless, under clinical suspicion of CAI, basal measurement of cortisol levels is recommended. In these cases, basal cortisol ≤ 88 nmol/L (3 μg/dL) confirms CAI, while 415 nmol/L (15 μg/dL) or more excludes CAI ( 19 , 20 ). Values between 88 nmol/L (3 μg/dL) and 415 nmol/L (15 μg/dL) will require dynamic tests to evaluate corticotroph axis integrity ( 20 , 32 ).

A cut-off level of basal cortisol that can differentiate between CAI or a normal corticotrophic axis would be beneficial to guide corticosteroid replacement. However, the literature on this topic is inconclusive. A meta-analysis including 13 studies suggested different cut-offs; cortisol <138 nmol/L (5 μg/dL) translates to >92% probability of CAI (95% CI: 75%-99%), whereas cortisol >359 nmol/l (13 µg/dL) translates to <9% probability of CAI (95% CI: 3%-18%) ( 33 ). A recent study using current laboratory assays (LC-MS and monoclonal antibody) described a baseline cortisol level of <55 nmol/L (2 μg/dL) related to an inadequate pituitary response to stimulus tests ( 31 ).

**Figure 1:**
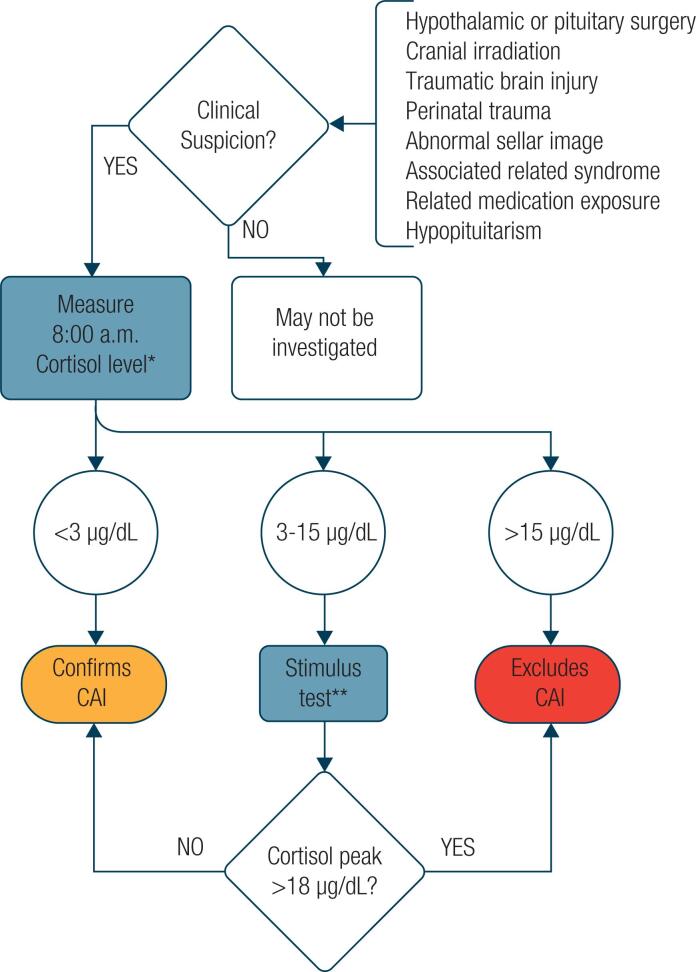
Suggested diagnostic flowchart for central adrenal insufficiency (CAI). *Notes on various laboratory assays. LC-MS and monoclonal assays have different cut-offs.** Attention to the cortisol assay and the kind of stimulus test. LC-MS: monoclonal assays and glucagon stimulus test have different cut-offs.

When the measurement of basal cortisol is insufficient, a pituitary stimulation test is necessary ( 1 , 2 , 20 , 32 , 34 ). Usually, a cortisol level greater than 497 nmol/L or 18 μg/dL (using polyclonal antibody assays) indicates a normal ACTH response and excludes CAI ( 1 , 2 , 20 , 32 ). Nevertheless, Javorsky and cols. suggested that a cortisol cut-off of 387 to 415 nmol/L (14 to 15 μg/dL) depending on the assay used (LC-MS/MS or monoclonal antibody) can exclude CAI ( 31 ). It is essential to highlight that none of the tests can be considered reliable for CAI diagnosis. Even the gold standard test (described above) is prone to false-positive results ( 1 , 2 ). Therefore, clinical evaluation is essential. We suggest the following flowchart for CAI assessment ( Figure 1 ).

Once the laboratory diagnosis of CAI is confirmed, an imaging evaluation of the pituitary is necessary (except in cases induced by opioids and glucocorticoids) to detect the presence of a tumor or other infiltrative processes ( 8 ). In addition, the status of other pituitary hormones must be assessed together with sellar MRI ( 8 ).

#### Stimulus tests

##### Insulin Tolerance Test (ITT):

ITT is the gold standard test. However, it is contraindicated in patients with high cardiovascular risk, seizures, pregnancy, cerebrovascular disease, and in those over 60 years of age. Samples were collected at 0, 30,60,90, and 120 min after the stimulus with intravenous insulin administration (0.05 0.15 U/kg). For adequate stimulation, a glucose level of <40 mg/dL is mandatory at any point after insulin administration ( 1 , 2 , 32 ). Potentially dangerous side effects such as neuroglycopenia should be kept in mind and the patients monitored.

##### ACTH Stimulation Test:

This test was used to verify adrenal responsiveness to ACTH using recombinant ACTH (Synacthen^®^ or Cortrosyn^®^) as a stimulus. However, this test works better after adrenal atrophy is established, which usually occurs approximately six months after pituitary injury. Thus, this test cannot be used for diagnosing acute CAI ( 1 , 2 ).

Currently, a dose of 250 µg of ACTH is largely recommended with samples collected at 0, 30, and 60 min after intravenous application. However, the short Synacthen^®^ test (1 µg) has a possible bias owing to the difficulty of preparing a meager ACTH amount, which runs the risk of administering inaccurate doses ( 32 ).

##### Glucagon Test:

Glucagon is an alternative test to predict pituitary response, especially in countries where ACTH is not easily available, such as in Brazil ( 35 ). This test was performed with intramuscular administration of 1 mg glucagon. Samples must be collected at 0, 30, 60, 90, 120, and 180 min ( 1 , 2 ). Several studies suggest a cut-off of 163-167 ng/mL (which corresponds to approximately 450-460 nmol/L or 16-17 μg/dL, respectively) to exclude CAI, with acceptable sensitivity and specificity ( 35 - 37 ). Although this test is longer used and is less accurate than ITT, it can be a good alternative to CAI diagnosis.

##### CRH Test:

This test is not used anymore, but it will be discussed to highlight its historical importance. This test consists of an intravenous administration of 100 µg of CRH and dosages of cortisol and ACTH at –5, −1, 0, 15, 30, 60, 90, and 120 min ( 1 , 2 ). A normal response is when ACTH is two to four times higher than baseline, generally occurring at 30 min, followed by a cortisol peak at 60 min ( 1 ). Unfortunately, CRH is not very affordable, and its clinical utility is questionable ( 27 ). This test differentiates secondary from tertiary adrenal insufficiency and could serve as an alternative for acute CAI diagnosis ( 1 , 2 ). However, a recent study ( 38 ) comparing basal cortisol versus a CRH test in the early postoperative period after trans-sphenoidal surgery showed that basal cortisol is sufficient to guide glucocorticoid replacement ( 38 ).

##### Overnight Metyrapone Test:

Metyrapone inhibits adrenal 11-beta-hydroxylase and the conversion of 11-deoxycortisol (11-DOC) to cortisol, thereby decreasing the negative cortisol feedback on ACTH ( 39 ). Despite a robust pharmacological rationale, the diagnostic accuracy of this test depends on the ACTH and/or 11-DOC cut-off used. The assays for 11-DOC are not easily available ( 9 , 40 ). Safety is another concern because this test carries a risk of adrenal crisis, and mistakes can occur from other drugs affecting metyrapone clearance ( 1 , 9 , 40 ). Metyrapone is also not available in Brazil nor in some other countries. These issues limit the widespread use of this test, and further studies are necessary to validate its clinical utility.

### Diagnosis future perspectives

This topic will discuss other laboratory approaches and tests that deserve attention, and current research focuses on improving CAI diagnosis.

#### Free Cortisol Dosages:

Cortisol binding globulin (CBG) binds cortisol with high affinity and low capacity ( 41 ). Besides hormone carriage, CBG has substantial importance in modulating cortisol release (depending on temperature, neutrophilic factors, and inflation markers of the target tissues) ( 42 ). Total serum cortisol is the most cost-effective assay for the initial evaluation of the HPA axis. However, in some cases, the free cortisol dosage is indispensable ( 42 , 43 ). False normal serum cortisol is observed when CBG is increased (due to pregnancy, oral contraceptive pills, and other medications) ( 40 ). The opposite is true. Lower CBG levels (due to critical illness or SERPINA6 polymorphisms) could result in CAI overdiagnosis ( 43 , 44 ).

Taking all this into account, free cortisol measurement, either directly in serum or saliva or indirectly using calculated values, is helpful in situations where the total cortisol value is incongruent with clinical presentation.

#### DHEAS:

Dehydroepiandrosterone sulfate (DHEAS) is exclusively produced in the adrenal reticularis zone by ACTH stimulus ( 1 , 2 ). Some studies have suggested that impairment of adrenal androgen secretion precedes glucocorticoid deficiency in patients with CAI ( 45 - 47 ). Its mechanisms are not fully understood, but some insights are presented by Topor and cols. in an *in vitro* study, supporting that CAI is extremely unlikely if the DHEAS level is normal ( 45 , 48 ).

DHEAS has a longer half-life (about 20 h) and lesser diurnal variation than cortisol ( 47 ), which can be advantageous. These two factors provide serum levels of DHEAS all day long (unlike cortisol). In contrast, DHEAS production varies based on sex and age, with lower levels in prepubertal children and the elderly. Charoensri and cols. suggested the DHEAS ratio (DHEAS level divided to 5th percentile of normal range to sex and age) ( 49 ). Using this ratio, they showed that a DHEAS ratio higher than 1.78 is a very sensitive marker of HPA integrity. Despite showing some potential to diagnose CAI, more research is needed to clarify its use in clinical practice.

## HOW?

### Management

Glucocorticoid replacement in CAI is challenging in many ways. First, it is difficult to mimic the circadian rhythm of cortisol secretion. Second, there are several options for oral glucocorticoid replacement, and third, there are no reliable biomarkers to guarantee appropriate doses.

The daily physiological secretion of cortisol by the adrenal gland is estimated to be between 5 and 10 mg of cortisol per m² surface area ( 50 ). Based on this, a dose of 15-20 mg/day of hydrocortisone equivalents (HCeq) in adults and 8-10 mg/m² of HCeq in children is estimated to be adequate for corticosteroid replacement ( 28 ).

There are several options for oral glucocorticoid compounds ( Table 3 ) ( 1 , 2 ). The guidelines recommend that hydrocortisone or cortisone acetate is divided into two or three doses per day with a larger dose early in the morning ( 20 , 32 ). If used in a three-times/day scheme, the latest dose in the afternoon needs to be close to 4-6 p.m. ( 9 , 28 ) since hydrocortisone administration in the evening and at night is related to insulin resistance and sleep disturbances ( 51 , 52 ). Medications with a shorter half-life, such as hydrocortisone and cortisone acetate, are preferred, owing to their ability to mimic nictemeral cortisol secretion at multiple doses. Cortisone acetate is a pro-drug with a slight delay in onset because it requires activation by hepatic 11β-hydroxysteroid dehydrogenase. Ekstrand and cols. showed worse body composition and metabolic profile outcomes in patients with CAI who changed from cortisone acetate to HCeq ( 53 ), which was reinforced by Filipsson and cols. ( 54 ).

**Table 3:** Synthetic glucocorticoids and their characteristics

	Hydrocortisone equivalent dose (HCeq)	Glucocorticoid activity	Mineralocorticoid activity	HALF-LIFE (hours)	KIND
Hydrocortisone	20 mg	1	1	8-12	Short action
Cortisone acetate	25 mg	0.8	0.8	8-12	Short action
Prednisone	5 mg	4.0	0.2	12-36	Intermediate action
Methylprednisolone	4 mg	5.0	0.2 to 0.5	12-36	Intermediate action
Prednisolone	5 mg	4.0	0.2	12-36	Intermediate action

Hydrocortisone and cortisone acetate are not widely available in some countries, such as Brazil. Consequently, many patients need to use intermediately acting glucocorticoids (prednisolone or prednisone). Even under physiological doses, this kind of glucocorticoid can induce unfavorable metabolic effects, as it impacts the circadian level of cortisol production ( 55 ).

In addition to drug choice, corticoid doses matter ( 54 , 55 ). There is a relationship between glucocorticoid dose and BMI, serum triglyceride, cholesterol, and low-density lipoprotein levels. Worse parameters were found in patients receiving more than 20 mg/day of HCeq ( 54 ). The same study showed that by reducing the HCeq dose by 50%, it was possible to improve body composition and lipid profile ( 54 ).

Deficiencies of other pituitary axes are common among individuals with CAI. They can modify corticosteroid bioavailability and impact the corticosteroid dosage. Patients with hypopituitarism undergoing growth hormone and/or estrogen replacement require higher glucocorticoid doses than those without these deficiencies ( 20 , 28 , 32 ). Glucocorticoid doses may also be influenced by residual ACTH secretion ( 28 ). This is particularly true among patients with partial ACTH deficiency, which can be either over- or mis-replaced by the recommended doses ( 28 ). While the overuse of glucocorticoids leads to unfavorable metabolic outcomes, suboptimal replacement can contribute to morbidity in CAI ( 28 ).

Some drugs can affect CYP3A4, the major enzyme in cytochrome P450, which metabolizes medications that affect the metabolism of synthetic corticosteroids. Therefore, glucocorticoid intake should be reduced with concomitant use of CYP3A4 inhibitors (antifungals and antiretrovirals); it should be increased with the use of drugs that activate CYP3A4 (mitotane, rifampicin, carbamazepine, topiramate, barbiturate, levothyroxine) ( 8 ).

Alternative corticosteroid formulations have recently been studied, with promising results (see future perspectives).

Nevertheless, more research is needed to identify biomarkers that enable personalized and physiological glucocorticoid replacement (see Future Perspectives). Without this kind of laboratory assessment, we can only reduce medication doses in the presence of signs of hypercortisolism without accounting for to subclinical adverse outcomes.

## SPECIAL ISSUES AND QUESTIONS

### Mortality and CAI

CAI occurs mainly in combination with multiple pituitary deficits. In hypopituitarism, mortality is associated with untreated growth hormone deficiency, untreated hypogonadism, and over-treated hypocortisolism ( 56 , 57 ). Indeed, the risk of death due to infectious disease between patients with hypopituitarism is 1.6-fold higher than that in patients with ACTH deficiency, probably due to adrenal crisis during an concurrent illness ( 56 ).

In a meta-analysis of 12 studies, Jasim and cols. showed that the main mortality determinants in hypopituitarism were female sex, young age at diagnosis, transcranial surgery, radiotherapy, craniopharyngioma, and diabetes insipidus ( 58 ). This systematic review excluded studies with ACTH-and GH-secreting adenomas and included papers published until 2015. Furthermore, in 2016, a prospective study, including 519 patients with non-functioning pituitary adenomas, showed a higher mortality rate among patients with ACTH and FSH/LH deficiency ( 59 ). Another study regarding long-term follow-up of patients with acromegaly reported higher mortality in patients with CAI ( 60 ).

Over-replacement and subclinical hypercortisolism may be responsible for the high morbidity and mortality rates in patients with CAI and hypopituitarism ( 59 ). Conversely, under-replacement can also induce adrenal crises and contribute to higher mortality rates ( 56 , 57 ).

### Patient education

Therapeutic patient education in CAI, as in many other chronic conditions, is a crucial issue. It is beyond simply transmitting information. Patients with CAI should be encouraged to acquire and maintain competencies that help them become more independent, to improve their quality of life (QoL), and to protect themselves from potentially life-threatening risks linked to their condition. Patients (besides learning activities to cultivate healthy habits and promote self-care) must be trained to maintain proper use of medications, port an emergency card, recognize situations that trigger an adrenal crisis, and act appropriately in case of adrenal crisis ( 61 ).

### Is there space for DHEA replacement?

Androgenic deficiency in women with CAI has been associated with lower quality of life (QoL) ( 62 ). This piece of evidence opens the discussion about the benefits of DHEA replacement in women with CAI. However, clinical studies on this topic are limited for several reasons (heterogeneous population, several QoL questionnaires, different doses, and treatment times). A double-blind, randomized crossover study evaluated the daily replacement of 50 mg DHEA in 24 women with either primary or secondary adrenal insufficiency for four months showed an improvement in depression/anxiety parameters and sexual function in those undergoing DHEA replacement ( 63 ). However, a meta-analysis including 10 studies showed only a small improvement in QoL scores and a slight decrease in the occurrence of depression. In the same study, the benefits related to anxiety and libido were not statistically significant ( 64 ).

Although *in vitro* studies ( 65 - 67 ) suggest that DHEA has a significant anti-atherosclerotic role, clinical study results ( 68 - 70 ) are still conflicting or insufficient to prove this benefit. The effects of DHEA on insulin resistance and bone metabolism remain unclear ( 1 , 8 , 70 ). Since DHEA can be converted to estrogen, its impact on thromboembolic risk and estrogen-dependent cancers is unclear ( 8 ). In conclusion, the current evidence points out the benefits of SDHEA replacement, but its recommendation requires more robust clinical evidence ( 8 , 71 ).

### Adrenal function and intensive care medicine

Critical illness-related corticosteroid insufficiency (CIRCI) is a clinical concept that describes the impairment of the hypothalamic-pituitary axis during organic stress in the intensivist context ( 3 ). The rationale for this concept is based on the sensitivity of the glucocorticoid receptor, which is highly variable in critically ill patients ( 42 , 43 ). Cytokines, chemokines, and bacterial toxins can interact with hypothalamic and peripheral receptors in an orchestrated manner to increase the availability of free cortisol in specific and strategic tissues. Nevertheless, a minor dysregulation in this metabolic response can provoke many adverse effects, as observed in cytotoxic storm syndrome ( 3 ). In septic shock, relative adrenal insufficiency is a marker of disease severity. However, its recognition is challenging from a clinical and laboratory point of view. Several current studies present controversial results regarding the dosage of cortisol or free cortisol in this context, both at the basal time and after a stimulus ( 3 , 72 - 74 ). Currently, it is not advisable to perform adrenal tests. The use of hydrocortisone is indicated in the context of septic shock refractory to vasoactive drugs ( 72 , 73 ).

### HPA axis recovery

Another exciting topic is HPA recovery. This recovery can occur, but it cannot be predicted after neurosurgery or prolonged exposure to synthetic corticosteroids.

In a retrospective study, Leong and cols. showed that 61% of patients recovered the corticotroph axis within two years after the interruption of corticosteroid therapy. Their data also showed that a basal cortisol level of 243 nmol/L (8.8 μg/dL) had a sensitivity of 70% and specificity of 93% in predicting adequate response to the short Synacthen^®^ test ( 75 ). Another study, using salivary cortisol and cortisone dosage, showed that the basal serum cortisol level is superior to salivary tests in predicting axis recovery after corticosteroid therapy ( 76 ).

The CRH test after pituitary surgery, although safe, does not demonstrate adequate accuracy in predicting long-term corticotroph function ( 38 ). Basal cortisol levels on the second or third postoperative day of less than 220 nmol/l (8 μg/dL) suggest that glucocorticoid replacement is necessary. ACTH axis recovery can occur up to 2 or 3 years after surgery. Repeating the hormonal evaluation re-evaluation followed by an ACTH stimulation test at least six months after surgery is suggested to test HPA recovery ( 1 , 2 , 38 ).

## FUTURE PERSPECTIVES

### Newer glucocorticoid replacement alternatives:

Medications with a modified release (Chonocort^®^) and dual release (Plenadren^®^) of hydrocortisone have been studied. Their pharmacokinetics promote corticoid bioavailability closer to that of circadian production ( 19 , 28 , 60 ). Plenadren^®^ is licensed in Europe. It is administered once a day to improve patient adherence. Besides comfortable posology, this drug has demonstrated benefits regarding body composition, metabolic profile, and bone safety ( 20 , 40 , 77 , 78 ). However, Plenadren^®^ has 20% less bioavailability than oral hydrocortisone, and dose adjustment is necessary ( 77 ). Although exciting, long-term clinical trials are expected to expand the evidence regarding the benefits of smart release hydrocortisone in CAI management.

### Animal model studies:

Besides having a broad analogy with human DNA, zebrafish is a well-established animal model for the study of adrenal diseases due to its diurnal habit. Animal studies with zebrafish, metabolomics, and transcriptomics provide exciting insights into the transcription of gene cycles and metabolomic profiles in models of primary and secondary adrenal insufficiency ( 79 , 80 ). We believe that these findings will provide exciting findings for further biomarker studies in humans. Basic research on CAI can also help find a way to individualize cortisol replacement. As a result of their investigative potential, more research should be conducted on this topic.

In conclusion, CAI is a life-threatening condition in which diagnosis and management are still challenging. A basal cortisol dosage is able to diagnose CAI, but it relies on a straight cut off. Therefore, several patients presented with a gray zone demanding stimulus tests. CAI treatment is based on glucocorticoid replacement, but many questions remain about its optimal posology. In the absence of a clinical biomarker of glucocorticoid replacement adequacy, there is a risk of under-or over-glucocorticoid replacement. It is urgent to determine how to diagnose CAI, differentiate partial from complete adrenal insufficiency, and clarify the role of new glucocorticoid replacement options in CAI management.
